# Speech Production Development in Mandarin-Speaking Children: A Case of Lingual Stop Consonants

**DOI:** 10.3390/bs15040516

**Published:** 2025-04-13

**Authors:** Fangfang Li

**Affiliations:** Department of Psychology, University of Lethbridge, Lethbridge, AB T1K 3M4, Canada; fangfang.li@uleth.ca

**Keywords:** speech acoustics, stop consonant, Mandarin, VOT

## Abstract

Lingual stops are among the earliest sounds acquired by young children, but the process of acquiring the temporal coordination of lingual gestures necessary for the production of stop consonants appears to be protracted. The current research aims to investigate the developmental process of lingual stop consonants in 100 Mandarin-speaking 2- to 5-year-olds using the acoustic parameter voice onset time (VOT). Children were engaged in a word-repetition task and recorded while producing words that begin with /t/, /d/, /k/, and /g/. Results indicate well-established contrasts between /t/ and /d/ as well as between /k/ and /g/ by age 2. However, comparing with adults’ speech patterns, children’s speech productions are characterized by greater within-category dispersion and overlap, as well as smaller phoneme discriminability. Mandarin-speaking children also go through an “overshoot” stage by producing longer-than-adult VOT values, especially for voiceless aspirated stops /t/ and /k/. Lastly, unlike adults who exhibit gender-specific patterns in VOT, boys and girls do not show distinct patterns in their VOT by age 5. These results will be discussed in relation to children’s lingual motor control development and the organization of phonological and phonetic structures during the process of language acquisition.

## 1. Introduction

Most children learn to produce speech sounds that form words and sentences during the first few years of their lives (Jakobson, 1941/1960). Lingual stop consonants, such as the sounds /t/ (as in *t*ea in English or as in *t*ī, 踢, in Mandarin), /d/ (as in *d*eer in English or as in *d*ī, 低, in Mandarin), /k/ (as in *c*ake in English or as in *k*ū, 哭, in Mandarin), and /g/ (as in *g*oat in English or as in *g*ū, 姑, in Mandarin), are sounds produced with the tongue making a complete obstruction in the oral cavity followed by a release of the closure and are among the earliest sounds acquired by young children due to the relative ease of articulation of these sounds (Rice & Avery, 1995; Vennemann, 1971). However, the process of acquiring the temporal coordination of lingual gestures necessary for the production of these consonants appears to be protracted (Hitchcock & Koenig, 2013; Lowenstein & Nittrouer, 2008; Macken & Barton, 1980). Most of the studies describing this process of articulatory coordination utilize voice onset time (VOT) as the acoustic measurement.

**Figure 1:**
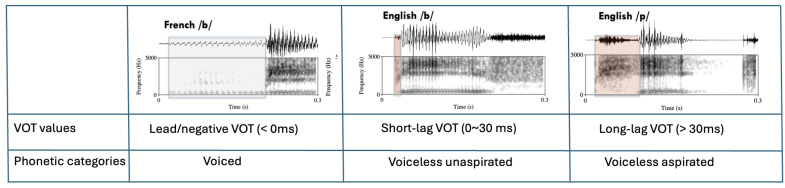
VOT values and corresponding phonetic labels, with spectrographic examples from English and French. English has two voicing categories in word-initial position: ‘voiceless unaspirated’ and ‘voiceless aspirated’ stops. French has the ‘voiced’ stop category. Pink shaded areas indicate positive VOT regions. Gray shaded area indicates negative VOT region.

VOT refers to the temporal difference between stop closure release and the initiation of vocal fold vibration accompanying the release during the production of a stop consonant (Lisker & Abramson, 1964). VOT has frequently been demonstrated to be the most effective acoustic parameter in differentiating phonological contrasts for stops in a variety of languages such as English, French, Mandarin Chinese, Korean, Dutch, Hindi, and many endangered languages (Caramazza & Yeni-Komshian, 1974; Cho & Ladefoged, 1999; Keating et al., 1983; Lisker & Abramson, 1964; Riney et al., 2007; among others). According to Cho and Ladefoged (1999) and Keating (1990), the majority of languages partition the VOT continuum into three voicing categories: “voiced” (<0 ms, lead VOT), “voiceless unaspirated” (0~30 ms, short-lag VOT), and “voiceless aspirated” (>30 ms, long-lag VOT), although the exact delineation of the VOT continuum and the associated phonological label for voicing categories are very much dependent on the individual language (see Riney et al., 2007 for Japanese; Cho et al., 2002, for Korean, and Kharlamov, 2022, for Russian). As an example, English has six stop consonants: /p, t, k/ and /b, d, g/. When these stop consonants are in word-initial position, they are characterized by having a two-way voicing contrast between long-lag and short-lag VOT for voiceless aspirated (i.e., /p/, /t/, /k/) and voiceless unaspirated stops (i.e., /b/, /d/, /g/) respectively (Keating et al., 1983; Zlatin, 1974). Mandarin is similar to English in that it also distinguishes stops with short-lag VOTs (voiceless unaspirated stops, /b/, /d/, and /g/) and long-lag VOTs (voiceless aspirated stops, /p/, /t/, and /k/). In some languages, such as French, a two-way distinction is made between “voiced” (prevoicing, lead/negative VOT) and “voiceless unaspirated” stops (short-lag VOT) (Caramazza & Yeni-Komshian, 1974; MacLeod & Stoel-Gammon, 2009). Other languages, such as Thai, contrast all three categories of stop consonants, delineating the VOT continuum into “lead/negative”, “short-lag”, and “long-lag” regions (Gandour et al., 1986; Kessinger & Blumstein, 1997) (Figure 1).

VOT has been used extensively as an objective instrumental tool in studying developmental processes and phonological universals for voicing contrast across the world’s languages (Cho & Ladefoged, 1999; Lisker & Abramson, 1964; Lowenstein & Nittrouer, 2008; Macken & Barton, 1980). There exists a wealth of studies utilizing VOT to investigate how children acquire word-initial stop consonants, most of which focus on the English language. These studies typically examine whether children at different ages are able to produce voicing contrasts needed to create phonological distinctions between /p/ vs. /b/, /t/ vs. /d/, or /k/ vs. /g/, and whether their phonetic implementation of the contrastive categories that mark these distinctions are in similar VOT ranges as those of adults.

The majority of previous studies indicate that children are able to produce differentiated VOT patterns for contrastive voicing categories of stop consonants in the first two or three years of life (see Hitchcock & Koenig, 2013, for a review on VOT studies on children before 3 years of age). However, these VOT values are not adult-like and continue to be refined in later childhood (Gilbert, 1977; Hazan et al., 2013; Whiteside et al., 2003; Zlatin & Koenigsknecht, 1976). Before 2 years of age, English-speaking children produce stop productions with VOT values falling primarily into the short-lag range for both voiceless unaspirated and voiceless aspirated categories, corresponding only to adults’ voiceless unaspirated stop category. Children then start to produce voiceless aspirated stops with longer VOT values to achieve a voicing contrast, but it will take a few more years for them to approach adult-like VOT (Hazan et al., 2013; Hitchcock & Koenig, 2013; Kent, 1976; Kewley-Port & Preston, 1974; Lowenstein & Nittrouer, 2008; Macken & Barton, 1980; Nittrouer, 1993; Zlatin & Koenigsknecht, 1976).

**Figure 2:**
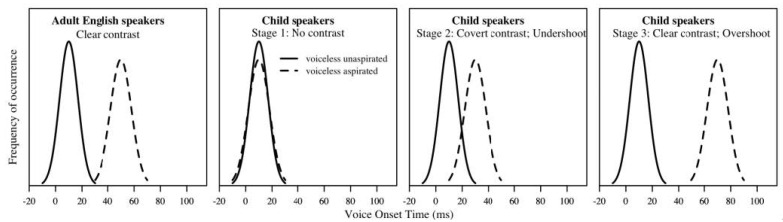
Hypothetical VOT curves for voiceless unaspirated stops vs. voiceless aspirated stops produced by English-speaking children at different stages as compared to adult speakers, based on Macken and Barton (1980).

In their classic study, Macken and Barton (1980) proposed a three-stage model of English-speaking children’s consonantal voicing development as measured by VOT, based on a longitudinal study of four English-speaking children, recorded biweekly from age 1; 6 (year; month) to 2; 6. Figure 2 schematically plots the VOT distributional curves for voiceless unaspirated stops vs. voiceless aspirated stops produced by adults and children at different stages, based on the findings of Macken and Barton (1980). As shown in Figure 2, in Stage 1, children produce no voicing contrast, and their VOT values form a unimodal distribution in the short-lag VOT range that corresponds to adults’ voiceless unaspirated category. In Stage 2, children produce “covert contrast”, which is a statistically reliable distinction in VOT values between voiceless unaspirated stops and voiceless aspirated stops, but the phonetic distance between the two voicing categories is much smaller compared to that of adults, and such a distinction is not conspicuous to adult listeners. This is a type of “undershoot” in articulation, since children’s phonetic implementation of the contrastive categories falls short of the canonical VOT values (if we assume that the target values are the ones produced by adults). Therefore, during this stage, children are able to distinguish the two voicing categories by forming a bimodal distribution that separates voiceless unaspirated stops from voiceless aspirated stops, but the range of VOT distribution for both voicing categories primarily occupies the short-lag range. In Stage 3, children go through an “overshoot” phase, in which they start to produce a voicing contrast with their mean VOT values longer than those of adults for voiceless aspirated stops. During this stage, children’s articulation is considered “overly” clear as the phonetic distance between the two voicing categories is greater than that of adults. After this stage, children gradually show shortened VOT values approximating adult norms.

According to the existing literature, children acquire stop consonants at a minimum of two levels of phonological representations. The first level is the level of **phonological contrasts**. The fact that most 2-year-olds are able to reliably distinguish voiceless unaspirated and voiceless aspirated stops (i.e., /t/ vs. /d/ and /k/ vs. /g/) suggests that they have started to establish the phonemic contrasts involved in stop consonants. The second level is about **phonetic learning** to approximate adult VOT values. The “undershoot” and then “overshoot” phases reflect the protracted process that children go through for phonetic learning of coordinating different articulators (i.e., larynx, vocal cords, lips, and tongue) to precisely time the amount of aspiration needed after the oral closure release during stop consonant productions. Many researchers believe that a critical aspect of phonetic learning is to gain motor control for temporal coordination of articulatory gestures (Nittrouer, 1993; Repp, 1986; Smith & Goffman, 1998). It is important to note that immature speech motor control, as characterized by imprecise coordination between articulators with respect to the timing of articulatory gestures, is also frequently reflected in larger intraspeaker variability. Indeed, measures of VOT (other than the mean values) frequently characterize children’s stop production as having greater within-category standard deviation and more between-category overlap, both of which are manifestations of children’s more variable and inconsistent speech production. Thus, the stabilization of stop production and the reduction in variability are important dimensions for the phonetic learning of phonological categories (Kent, 1976; Koenig, 2001; Lowenstein & Nittrouer, 2008).

### 1.1. Developmental Research on Children’s VOT Production in Other Languages

Research on children’s VOT productions in languages other than English is less common and has mainly been focused on languages that have different types of voicing contrast from English (i.e., Eilers et al., 1984, on Spanish; Gandour et al., 1986, on Thai; Davis, 1995, on Hindi; Allen, 1985; and MacLeod, 2016, on French; Brinca et al., 2016, on European Portuguese; Okalidou et al., 2010, on Greek; and Kong et al., 2012, on Greek and Japanese). For example, Gandour et al. (1986) examined VOT in children acquiring the three-way contrast in Thai, with VOT falling in “lead”, “short-lag”, and “long-lag” regions. They discovered that, similar to English-speaking children, Thai-speaking children initially produce all three categories of stop consonants in the short-lag VOT region without clear distinctions. These children then gradually develop the long-lag VOT category at 3 years of age but fail to properly produce the voiced category with negative VOT even at age 5. Meanwhile, considerable overlap was found between categories even if children’s means VOT values approximated those of adults.

In a study on French-speaking children aged 2; 4 to 6; 0, MacLeod (2016) found that 2-year-olds were able to produce distinctive voiced stops and voiceless unaspirated stops in the lead VOT and short-lag VOT range, respectively. Furthermore, although considerable variability was observed in both ranges, children were more variable in their productions in the lead VOT than in the short-lag VOT category.

Okalidou et al. (2010) compared the development of VOT in two dialects of Greek, Standard Greek and Cypriot Greek, that differ in the number of contrastive voicing categories. Standard Greek, like French, has a two-way contrast between lead and short-lag VOT, while Cypriot Greek is similar to Thai and contrasts lead, short-lag, and long-lag VOT. Their results indicate that children speaking Standard Greek mastered the two-way contrast earlier, at around 2; 6, than those speaking Cypriot Greek, who acquire the three-way contrast around 3 or 4 years of age. It was also found that children who speak Standard Greek have already distinguished lead and short-lag VOT by age 2, but their short-lag VOT values occupy a greater range than those of adults, and those values decrease over time, but the duration for lead VOT increases over time. This research suggests that it is easier to master a two-way voicing contrast than a three-way contrast. In addition, regardless of the number of contrastive categories, the process of phonetic implementation of these categories will continue much longer after children have learned to distinguish the categories.

The above-mentioned research on VOT development in different languages has yielded valuable insights into phonological universals regarding the acquisition of voicing contrast. First, children are able to make distinctive use of stop consonants by the age of 2, and by 3 years of age if they acquire a two-way contrastive system (such as English that contrasts voiceless unaspirated stops /b, d, g/ and voiceless aspirated stops /p, t, k/). Second, the short-lag VOT range seems to be the anchor category from which other contrastive categories emerge. Finally, the process of gradual reduction of categorical overlap and fine-tuning of categorical boundaries is also common to child speakers of different languages.

However, due to the diversity of types of contrasts in the languages reported, some issues remain unresolved. One such issue concerns the “overshoot” phenomenon first observed by Macken and Barton (1980) in English-speaking two-year-olds and then corroborated by a longitudinal study by Hitchcock and Koenig (2013), which tracked 10 English-speaking two-year-olds’ VOT production sampled biweekly for four months. Both studies noted an “overshoot” phase in all children observed, in which children produce exaggerated VOT values for voiceless aspirated stops, resulting in a greater mean VOT distance between the voiceless unaspirated and voiceless aspirated stop categories. Moreover, Hazan et al. (2013) examined the between-categories distance on English-speaking adolescents aged 11 to 14 years old and found that the mean VOT distance between voiceless unaspirated and voiceless aspirated stops in adolescents is longer than the between-category mean VOT distance of adults, suggesting that “overshoot” continues well beyond school years.

This “overshoot” phase is seldom reported in languages other than English. Since the majority of past research on languages other than English examines the acquisition of voicing contrast between lead VOT and short-lag VOT, a different type of contrast from the one in English, it is unclear whether this “overshoot” stage is a developmental phase unique to the English language or can also be attested to in children who speak other languages that have a similar type of voicing contrast to English.

### 1.2. Developmental Research on VOT in Mandarin Chinese

The current study reports VOT development in children aged 2; 0 to 5; 0, who speak Mandarin Chinese. Mandarin Chinese is a language with a two-way distinction between short-lag and long-lag VOT, similar to English (Chao & Chen, 2008; Chen et al., 2007; Rochet & Fei, 1991). Mandarin Chinese could serve as a testing case of whether “overshoot” is a common developmental stage for children learning this type of voicing contrast.

Developmental research on VOT in Mandarin-speaking children is scant. One recent study by Ma et al. (2017) examined VOT for a total of 85 children and adolescents aged 6 to 18 years. It was found that six- to seven-year-olds produced longer VOT than older children and adults for the voiceless aspirated stops, which suggests that Mandarin-speaking children may also undergo an “overshoot” stage. A second study by Yang (2018) examined 29 Mandarin-speaking three- to six-year-olds and 12 adults. Yang (2018) noted that young Mandarin-speaking children followed a similar VOT developmental pattern to that described by Macken and Barton (1980), first exhibiting a unimodal distribution of VOT for both voiceless unaspirated and voiceless aspirated stops and then splitting into a bimodal distribution. Unfortunately, the phenomenon of “overshoot” was not clearly identified in Yang (2018). Although “overshoot” was reported in Ma et al. (2017), the youngest children examined were 6-year-olds. Therefore, it is unclear when the “overshoot” stage begins to emerge. The current study aims to bridge this gap by testing children younger than age 6, in order to gain a deeper understanding of the developmental trajectory of VOT in Mandarin-speaking children’s stop consonant production.

It is also worth noting that adult speakers of Mandarin Chinese have been shown to exhibit sex-specific VOT patterns (Li, 2013; Ma et al., 2017; Peng et al., 2014). When producing lingual stops, female speakers distinguish voiceless unaspirated stops from voiceless aspirated stops with greater acoustic distance than males. This greater acoustic distance in women’s speech is achieved primarily by lengthening VOT for the voiceless aspirated stops. As of yet, it has not been determined whether young children learn to produce such sex-related speech patterns while they acquire voicing contrasts or after they have established their phonological categories. The answer to this question could shed light on the organization of phonological structures during development.

In the phonological acquisition theory proposed by Beckman et al. (2007), Munson et al. (2005), and Pierrehumbert (2003), the organization of phonological knowledge is composed of layers that are hierarchically structured, in which *socio-indexical knowledge*, such as sex-related speech patterns, is regarded as peripheral to the “core” *meaning-related categorical (*i.e., *lexical*) *knowledge* of sounds. The current study provides an opportunity to reveal possible connections between these two types of phonological knowledge and their relative order and timing of acquisition during development. The 2017 VOT study by Ma et al. also evaluated sex differences for VOT in their sample population of children older than age 6. They found no sex-related difference in VOT production until after 14 years old. Their results suggest that these two types of phonological knowledge may be acquired sequentially, which is also the premise adopted for validation/investigation in the current study.

### 1.3. Purposes and Predictions

The purpose of the current study is two-fold. First, it is to describe phonological and phonetic development of lingual stop production as measured by VOT in young Mandarin-speaking children aged 2 to 5 years old. At the phonological level, it is to identify when children are able to distinguish the two voicing categories robustly using VOT. At the phonetic level, the process of children approaching adult VOT norms will be examined to determine whether the phenomenon of “overshoot” exists in Mandarin-speaking children. Second, the study will look for possible sex-related differences in VOT in children of different age groups.

Based on previous studies, it is predicted that Mandarin-speaking children will acquire the voicing contrast around 2 to 3 years of age. It is also predicted that the phase of “overshoot” will appear shortly after the contrast is established. Also, based on Ma et al. (2017), it is predicted that children acquire the meaning-based sound category prior to their acquisition of socio-indexical categories (i.e., sex associated with VOT differences).

### 1.4. “Robustness of Contrast” Measures of Phonological Development

A growing body of research has demonstrated that a single measure of mean VOT is insufficient to capture the complexity of phonological development in children (Bailey & Haggard, 1980; Zlatin & Koenigsknecht, 1976). The most widely practiced approach in assessing whether or not English-speaking children have acquired the voiceless unaspirated vs. aspirated contrast is to conduct tests such as ANOVA that compare means of VOT values for sounds such as /t/ vs. /d/ (Lowenstein & Nittrouer, 2008; Koenig & Lucero, 2008). A significant mean difference would be interpreted as children having learned to produce distinct stop categories. However, we are also interested in how well children can make the distinctions between stop categories. As mentioned earlier, children’s speech productions can be variable, which results in VOT distributions of varying spread. Consider a case of two children who have similar mean VOT values for /t/ vs. /d/. Child 1 produces the sounds more variably than child 2, resulting in bigger VOT spreads for both sounds. Although both children are capable of distinguishing /t/ from /d/, clearly child 2’s category formation is more “robust”, since child 2 has learned to produce these sounds more consistently than child 1.

Another consideration is the degree to which two VOT distributions overlap for contrastive categories, which is not necessarily related to how compact or diffuse the shapes of VOT distributions are. For example, a child who produces /t/ and /d/ with wider spreads in VOT distributions but no overlap between the two distributions is considered to have established a more robust contrast than a child who produces these two sounds with a substantial overlap between the two VOT distributions that are compact in shape.

The current study adopts a set of measures proposed by Holliday et al. (2015) that include acoustic measures using VOT. These measures incorporate both the mean and spread of the VOT distribution, as well as logistic regression modeling, to assess the degree of category overlap. Collectively, these measures will help to quantify the robustness of the voicing contrast in Mandarin-speaking children at different ages and yield further insights into the full scope of the developmental process (Hazan et al., 2013; Hitchcock & Koenig, 2013) of how Mandarin-speaking children construct stop consonant categories.

The first measure, *Percentage of Correctly Predicted Productions* (*%CP*), assesses the degree of between-category overlap in the distribution of acoustic measurements between two contrastive categories such as /t/ vs. /d/. *%CP* calculates the percentage of a child’s productions that are correctly predicted by a mixed logistic regression model over a dataset containing information about acoustic measurements for a group of children’s speech productions. This measure was built on Newman et al.’s (2001) study, which suggests that between-categories overlap is a better predictor of consonant intelligibility than within-category dispersion or between-categories distance. Holliday et al. (2015) then designed *%CP* as an independent measure unaffected by dispersion or distance to assess the robustness of two contrastive voiceless sibilant fricative categories in English, /s/ vs. /ʃ/, in children’s speech. Specifically, the measure calculates the percentage of children’s productions that can be correctly predicted by a mixed logistic regression model that has the dependent variable being /s/ vs. /ʃ/, and the independent variables being frequency of fricative sounds produced by the children and their ages.

The remaining three measures were originally proposed by Romeo et al. (2013) and Hazan et al. (2013) to evaluate the internal structure of fricative place contrast and stop voicing contrast, as well as the perceptual consequences of the contrasts on intelligibility in word-initial tokens produced by adults and children aged 9 to 14 years old. Measures of *within-category dispersion* and *between-categories distance* were used to evaluate the degree of intra-talker variability by describing the distributional shape of contrastive categories and the relative distance between the two categories, based on the means of their distributions. Therefore, *within-category dispersion* is defined as the mean of the standard deviation of two contrastive categories (such as /s/ vs. /ʃ/ or /t/ vs. /d/); and *between-categories distance* is calculated by taking the difference between the means of the two categories. Lastly, *the discriminability score*, calculated based on signal processing theory (Stanislaw & Todorov, 1999), is defined as the between-categories distance divided by the square root of the mean of the variances of the two distributions. It is used to determine perceptual distance by taking potential bias into consideration. This measure was originally proposed to identify a listener’s perceptual sensitivity between two contrastive categories such as /s/ vs. /ʃ/, based upon results of perception experiments that ask listeners to make a forced-choice decision between two sound categories upon listening to a sound snippet produced by an adult or a child. The measure of *the discriminability score* was then adopted by Holliday et al. (2015) to apply to children’s fricative production data as a way to measure how discriminable two fricative category distributions are in children’s productions, which could also shed light on the degree to which listeners might perceive those productions as clear and separate categories.

The four measures just described have been demonstrated to correlate well with listeners’ perceptual judgments of speaker intelligibility and category-goodness ratings of English- and Japanese-speaking children’s voiceless sibilant fricative productions (Hazan et al., 2013; Newman et al., 2001). In this article, these four measures will be applied to Mandarin children’s stop productions for the first time to enable a more comprehensive evaluation of these children’s phonological development in VOT. It is worth noting that the *between-categories distance* measure is also well suited for investigating the issue of “overshoot”. If Mandarin-speaking children are similar to English-speaking children, then it is expected that the *between-categories distance* for children will first increase to become longer than adult values and then decrease to the adult norms.

## 2. Materials and Methods

### 2.1. Participants and Task

A hundred children aged 2 to 5 years old (see Table 1 for age and gender breakdown of the child participants) were recruited from a daycare center in Songyuan, Jilin Province, China. All children tested were residents of the city of Songyuan and were monolingual speakers of Mandarin Chinese. They all passed an otoacoustic emission (OAE) test for hearing screening at the frequency levels of 1000 Hz, 2000 Hz, 3000 Hz, 4000 Hz, and 5000 Hz prior to the testing. Also, they exhibited no previous speech, hearing, or language problems according to teacher and parental reports.

During the test, children were seated in front of an IBM laptop computer and were instructed to play a computer game by repeating each word after the computer. On the computer screen, there is an image of a duck climbing a ladder on the left margin and a picture for the test word in the center (see Appendix A for the image of the screen). Whenever a child repeated a word, the author pressed the space bar on the keyboard to move to the next picture/word, and the duck climbed up one step. Children were encouraged to help the duck to climb to the top of the ladder by repeating all the words they heard. Children’s speech productions were recorded through a Shure dynamic unidirectional microphone into a Marantz 660 digital recorder. The recording was made with a 44.1 KHz sampling frequency and 16-bit digitization.

Children generally appeared to enjoy the task and had no trouble completing it. The audio prompts were created by recording the author speaking the target words in child-directed speech. The whole word list contained words beginning with various lingual consonants, including stops, fricatives, and affricates, from a cross-language project on phonological acquisition in children (Edwards & Beckman, 2008). However, the current paper focuses on the analysis of lingual stops only, which includes /t/, /d/, /k/, and /g/ in front of the vowels /i/, /a/, and /u/. For each stop consonant, two words were elicited for each vowel context, with the exceptions of /k/ and /g/ in front of vowel /i/, which do not exist in Mandarin Chinese. Stimulus words were chosen to ensure that they could be represented with pictures that are culturally appropriate and familiar to young children (see Appendix B for the list of word stimuli for lingual stops). A total of 2020 words were elicited from all the child participants.

Data of 20 adults (18–30 years old, gender-balanced) reported in Li (2013) were reanalyzed to compare with children’s data. These adult participants were engaged in the same task as children and therefore can serve as a reference to gauge children’s speech production.

### 2.2. Measurements and Analysis

Children’s speech productions were segmented, and speech events, such as stop bursts and voicing onsets, were labeled using the speech software, Praat (Boersma & Weenink, 2005). Specifically, the burst onset was marked at the peak of the first spike of a cluster of transient noise that constitutes the stop burst. The voicing onset was marked as the first deviation of periodic glottal pulse. VOT was measured by calculating the time difference between the stop burst and the voicing onset. Acoustic measurements in Praat were made by two research assistants trained by the author. The two research assistants calibrated their measurements and reached a 99% degree of agreement before they started the actual VOT tagging. The author then trained a third research assistant who randomly selected 10% of the data, and the inter-rater reliability values between the third and the other two research assistants were 98% and 99%, respectively. The measurement error between the third and each of the two research assistants was within 1 ms.

Two types of analyses were conducted to assess the degree to which children acquire different aspects of voicing contrast for each place of articulation in Mandarin lingual stops. First, for each pair of lingual stops (i.e., /t/-/d/ and /k/-/g/), a three-way repeated measures ANOVA (target consonant × age × sex) was conducted to determine whether children were able to utilize VOT to distinguish /t/ from /d/ (or /k/ from /g/), and whether their ability to associate VOT with place distinction is different across age groups and between the two sexes. Second, for each child, his/her phoneme boundary was first calculated by taking the difference between the means of /t/ and /d/ (or /k/ and /g/) to determine how children at different age groups demarcate their VOT range for contrastive voicing categories.

Following the initial analyses, the four measures of robustness of phonological contrast (Holliday et al., 2015) were obtained to chart VOT development and to gauge the effect of intra-talker variability on the establishment of contrastive voicing categories. The definitions and interpretations of these four measures are provided in Table 2. Specifically, the measure *%CP*, which evaluates the degree of the overlap between the two VOT distributions (i.e., /t/-/d/ or /k/-/g/), is the likelihood of correct identification of each stop consonant in a sampled production dataset. Since this is an independent measure unaffected by dispersion or distance, in cases where children have two completely separable distributions for voiceless unaspirated and voiceless aspirated stops, *%CP* should be 100% despite how compact or diffuse the distributions are. Consequently, the higher the value of *%CP*, the smaller the amount of overlap that exists between the two distributions of contrastive categories. The measures of *within-category dispersion* and *within-categories distance* are independent from each other as well—a child producing two stop consonants (such as /t/ vs. /d/) that have a greater distance in the mean VOT values between the two distributions does not entail compact or diffuse shapes of these two distributions. Conversely, children can produce two categories with varying shapes of distributions despite the mean VOT distance between the two category distributions. All calculations and statistical analyses were conducted using R (R Development Core Team, 2011).

## 3. Results

### 3.1. Age-Related Difference in VOT Means and Distributions

A three-way repeated measure ANOVA for the /t/-/d/ contrast was first conducted, with the dependent variable being the VOT values for all the /t/ and /d/ tokens produced by 100 children, and the independent variables being the target consonant (/t/ vs. /d/), children’s age group (2-, 3-, 4-, and 5-year-olds), and children’s sex (male vs. female).

At the group level, a main effect of age was found to be significant (*F* (1, 92) = 17.29, *p* < 0.001, partial η^2^ = 0.03), suggesting that there are age-related differences in their VOT production of alveolar stops (/t/ and /d/). At the individual level, a main effect of target consonant was found to be significant (*F* (1, 96) = 6.42, *p* < 0.001, partial η^2^ = 0.56), which indicates that children in general are able to utilize VOT to distinguish /t/ from /d/. Post hoc Tukey HSD test revealed that all four age groups are able to produce /t/ and /d/ using significantly different VOTs (*p* < 0.001 for all four age groups). Finally, an interaction between target consonant and children’s age was found to be significant (*F* (1, 96) = 6.26, *p* = 0.01, partial η^2^ = 0.01). This significant interaction term suggests that VOT patterns differently according to children’s age. That is, the mean VOT for /t/ and /d/ varies depending on the specific age group.

**Figure 3:**
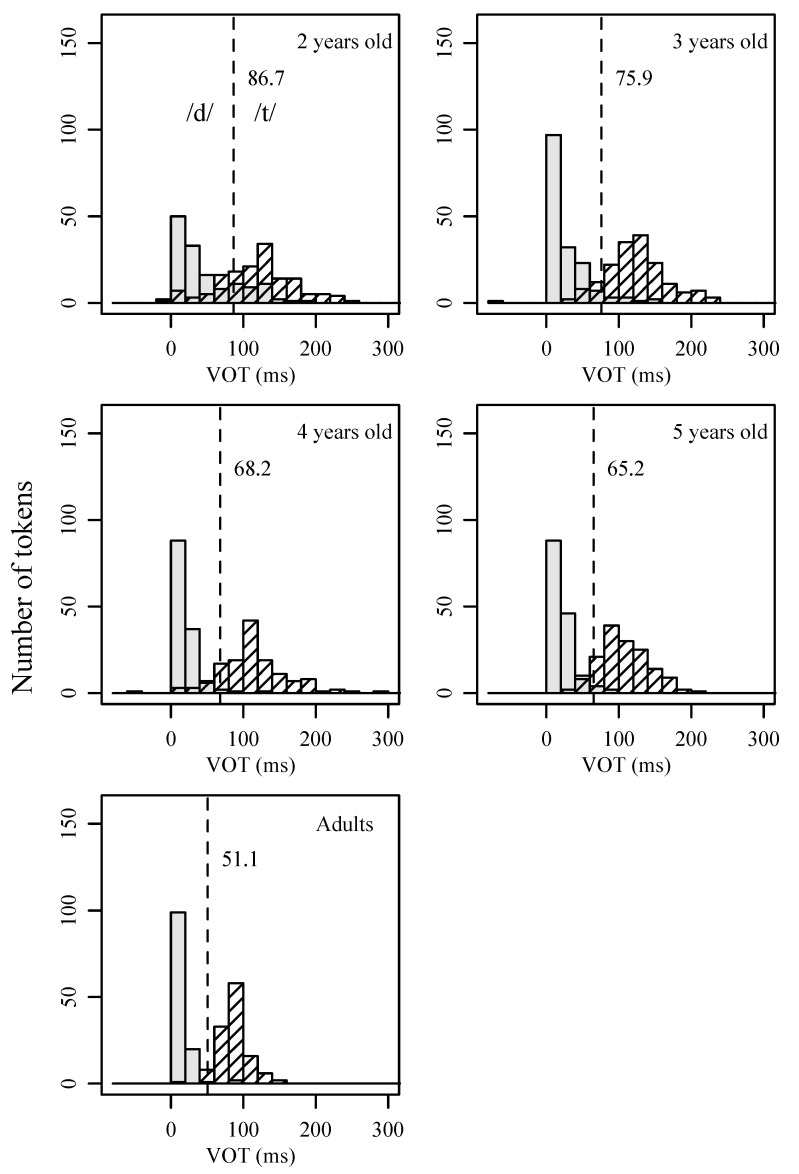
Histograms of VOT for alveolar stops /t/ and /d/ produced by children of different age groups and by adults. The dotted line indicates the phoneme boundary between /t/ and /d/, as calculated by taking the average of the mean of the VOT distribution for the two sounds.

This age-related variation is shown in Figure 3 and Table 3. Both the mean and the distribution of VOT differ substantially across age groups. This is especially true for two-year-olds. When comparing with older children, the 2-year-olds’ mean VOT for /d/ is larger, and their VOT distributions for both /t/ and /d/ have a greater spread, covering a wider range. The distributional shape for the two categories becomes more compact as children’s age increases. In addition, as shown in Figure 3, the phoneme boundary, as calculated by taking the average of the mean of /t/ and /d/, is highest in two-year-olds (86.7 ms) and decreases over time. By adulthood, the phoneme boundary becomes the shortest (51.5 ms).

**Figure 4:**
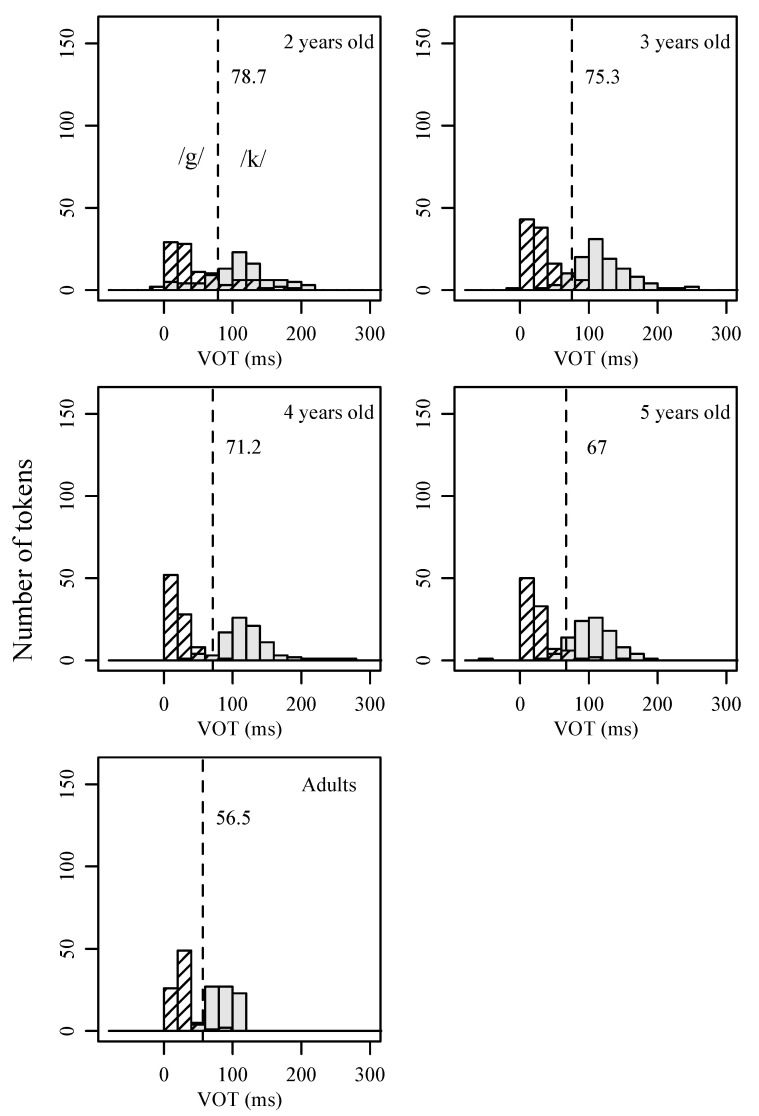
Histograms of VOT for /k/ and /g/ produced by children of different age groups and by adults. The dotted line indicates the phoneme boundary between /k/ and /g/, as calculated by taking the average of the mean of the VOT distribution for the two sounds.

The results of the three-way repeated measures ANOVA for the velar stops /k/ and /g/ are similar to that of the /t/-/d/ contrast. At the group level, a main effect of age was found to be significant (*F* (1, 92) = 5.98, *p* = 0.02, partial η^2^ = 0.01), which suggests that children at different age groups produce VOT between /k/ and /g/ in significantly different ways. The small effect size needs to be noted, however, indicating that the degree of differentiation is small. At the individual level, a main effect of target consonant was found to be significant (*F* (1, 96) = 6.42, *p* < 0.001, partial η^2^ = 0.56), indicating that children in general are capable of distinguishing voiceless unaspirated and voiceless aspirated velar stops. An interaction between target consonant and children’s age was found to be significant (*F* (1, 96) = 4.72, *p* = 0.03, partial η^2^ = 0.01), which suggests that each age group varies their VOT in contrasting the two stop consonants in specific ways. As shown in Figure 4, younger-aged children produce /k/ and /g/ with higher average means and greater average spreads. The phoneme boundary is highest in VOT value for two-year-olds and decreases gradually over successive age groups.

### 3.2. Sex-Related Difference in VOT Produced by Adults and Children

**Figure 5:**
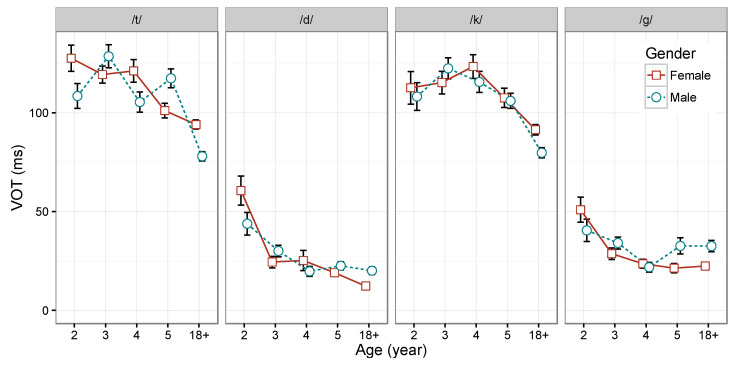
Mean VOT produced by children of different age groups and by adults, separated by speaker sex. Error bars indicate one standard error. Adult data were plotted as the last group in each plot with a label of 18+.

Two separate repeated measures ANOVAs were constructed for adults, one for each pair of stop consonants (i.e., /t/-/d/ and /k/-/g/). These ANOVA models were used to replicate the sex difference reported in Li (2013) and further serve as the reference data to be compared with the children’s productions. For each ANOVA model, the dependent variable was VOT values (in milliseconds), and the independent variables were target consonant, speaker’s sex, and the interaction between the two variables. In both ANOVA models, a significant interaction term was found between target consonant and speaker sex (*F* (1, 18) = 18.1, *p* < 0.001, partial η^2^ = 0.13, for the /t/-/d/ contrast; *F* (1, 18) = 17.6, *p* < 0.001, partial η^2^ = 0.12, for the /k/-/g/ contrast). The lack of an overall sex effect and the significant interaction term suggest that speaker sex does not affect the two contrasting consonants in the same way. The interaction between target consonant and speaker sex can be best illustrated by examining the VOT patterns in Table 1. Compared with adult males, adult females produce /t/ with longer VOTs and /d/ with shorter VOTs, resulting in greater separation between /t/ and /d/. Likewise, the categorical distance and the degree of separation between /k/ and /g/ are greater for adult females than for males. This reversed VOT pattern in the production of voiceless unaspirated and voiceless aspirated stops between the two sexes in adults is also clearly shown in Figure 5.

In contrast, results of the repeated measures ANOVAs for the two pairs of stop consonants produced by children showed no significant difference related to sex (*p* > 0.05 for the effect of sex, the interaction between sex and target consonant, and the interaction between sex, target consonant, and age, for both /t/-/d/ and /k/-/g/). This lack of sex-related difference in children is evident in Table 1 and Figure 5. Unlike adults, children show no consistent difference between the two sexes in the production of /t/-/d/ contrast (or /k/-/g/ contrast) in any of the age groups examined. These results suggest that although children are able to make lexical-based phonological contrasts, they have not acquired the sex-related difference by age 5.

### 3.3. “Robustness of Contrast” Measures and the Issue of “Overshoot”

Table 4 lists the results of the “robustness of contrast” measures using VOT for children and adult speakers of Mandarin. To calculate the first measure, *%CP*, two mixed-effects logistic regression models were constructed, one for adults and one for children. For each model, the dependent variable was /t/ vs. /d/ (or /k/ vs. /g/), and the independent variable was VOT (and age in the children’s model). A random intercept was built for each speaker, and each VOT entry was nested under individual speakers. The results indicate that adult models are able to predict with higher than 98% accuracy for the two pairs of contrast, thus achieving a *%CP* value higher than 98%. In contrast, the child models can only predict about 80% correctly for two-year-olds, with *%CP* being 79% for the /t/-/d/ contrast and 80% for the /k/-/g/ contrast. The values of *%CP* increased in older age groups of children. Since *%CP* is a measure of the degree of category overlap, these results demonstrate that adults produce voiceless unaspirated and voiceless aspirated stops with minimal overlap, while children (especially two-year-olds) exhibit greater overlaps between contrastive categories. The results are consistent with what has been depicted by Figure 3 and Figure 4 with respect to VOT distribution and the degree of overlap between voiceless unaspirated and voiceless aspirated categories.

For the second “robustness of contrast” measure, *within-category dispersion*, two-year-olds show the highest value, indicating the widest spread for the two consonant categories. As expected, adults show the smallest values, which suggests minimal degrees of dispersion. Similar to the developmental trend for *%CP*, a linear change from two-year-olds to adults was found for *within-category dispersion*. That is, the value of the dispersion decreases consistently as children’s ages increase. These results are in line with what Figure 3 and Figure 4 demonstrated for the VOT distributions of each stop consonant in children’s productions.

**Figure 6:**
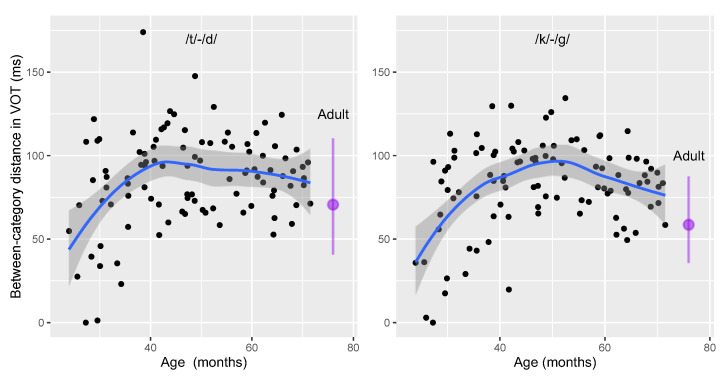
The relationship between voiceless unaspirated-aspirated category distance and children’s age. Each data point is the between-categories distance for one child. The blue line represents the best-fitted line, and the gray band indicates the 95% confidence interval. For each graph, the purple vertical line is the range of *between-categories distance* observed in adults, with the dot indicating the mean VOT value for adult speakers.

The third measure, *between-categories distance*, reveals an interesting nonlinear pattern: the distance is shortest in two-year-olds, enlarges in three- and four-year-olds, and then becomes shortened in five-year-olds. In addition, when comparing with adults, most children produce greater *within-category distance* for the two pairs of stop consonants. This nonlinear pattern of development agrees nicely with the definition of “overshoot”, where children, after learning to distinguish between two contrastive sounds, produce greater-than-adult categorical separation between the two sound categories and gradually approach adult norms afterwards. In order to further investigate the phenomenon of “overshoot”, the *between-categories distance* of each individual child was plotted against their age for each pair of contrasts (Figure 6). Furthermore, a best-fitted line and 95% confidence interval band were created for each contrastive pair (/t-d/ and /k-g/) using local polynomial regression in R, a non-parametric approach that fits multiple regressions in a local neighborhood for optimal smoothing span (Cleveland & Grosse, 1991). The best-fitted curves help to identify the developmental trend in children’s speech development. As shown in Figure 6, children initially expanded the *between-categories distance* as their age increased from 2, 0 to 4, and at 0. This category distance reaches its peak at 42 months for the /t/-/d/ contrast and at 48 months for the /k/-/g/ contrast. After that, the distance was shortened gradually over time but did not reach adult-like values by age 5.

The fourth measure, *phoneme discriminability score*, shows a linear increase over successive age groups. It reaches its peak at adulthood, indicating that children’s ability to produce distinct categories is enhanced over time. This result is interesting as the *phoneme discriminability score* was calculated based on *between-categories distance*, yet it does not exhibit the same pattern of “overshoot” as the *between-categories distance* measure. The different result could be due to the fact that the *phoneme discriminability score* was calculated by taking into consideration the within-category dispersion. Although children exhibit greater *within-category distance* than adults, their within-category dispersion is also greater than adults. Thus, children’s phoneme discriminability does not score better than adults. This result suggests that although children are able to separate the two categories to a greater extent than adults, this separation does not lead to greater distinction due to children’s larger within-category variability.

## 4. Discussion

The current research examines age- and sex-related stop production patterns in children aged 2 to 5 years who speak Mandarin Chinese. Mandarin Chinese, despite its large speaker population, has not been subjected to extensive studies previously with regards to stop consonant productions. VOT, the acoustic measure to describe phonological and phonetic acquisition of stop consonants, was utilized to reveal greater phonetic/articulatory details during the course of stop acquisition. This research has resulted in three major findings.

Firstly, at the phonological level, the results of repeated measures ANOVA indicate well-established phonological contrasts in children as young as 2 years old. At the phonetic level, children (especially those in the younger age groups) produce both voiceless unaspirated and voiceless aspirated categories with higher means and greater spreads, which push their phoneme categories to a higher VOT range. As children’s ages increase, the VOT distributions for the four stop consonants become more compact, and the phoneme boundaries are also reduced to smaller values, closer to but still not the same as adult norms. Previous studies of English consistently report the completion of the establishment of contrasts by age 2 (Macken & Barton, 1980; Lowenstein & Nittrouer, 2008; Zlatin & Koenigsknecht, 1976; also see Hitchcock & Koenig, 2013 for a review). Despite differences in fine phonetic details, particularly with respect to the actual mean and distributional shapes of VOT, these results, for the most part, are consistent with previous research in English. For example, greater within-category dispersions have been noted in 2-year-olds’ production in Zlatin and Koenigsknecht (1976) and Lowenstein and Nittrouer (2008), as well as in adolescents’ production reported in Hazan et al. (2013). The protracted process of acquiring adult-like VOT values in both English- and Mandarin-speaking children suggests that it may take many years for a child’s motor control system to mature sufficiently in order to learn the complex oral motor coordination required for speech production, regardless of the language that a child speaks. Stop consonant production requires three physiological mechanisms: first, articulatory movement to allow for oral closure and release; second, articulatory action to raise the velum to isolate the nasal cavity from the oral cavity; and third, articulations to initiate vocal cord vibration (Whiteside & Marshall, 2001). Children typically acquire motor control of the first two types of mechanisms early in life. It is the third mechanism, in addition to the motor coordination of different types of articulatory activities, that takes longer to develop. In fact, the long developmental time course for fine motor learning has been one of the most consistent findings in the literature of speech motor control (Smith, 2006; Smith & Zelaznik, 2004; Walsh & Smith, 2002). It has been found that the development of the capability to consistently coordinate oral motor patterns persists well into adolescence.

Secondly, no clear sex-specific VOT patterns were observed in children up to age 5. This finding is expected in light of Ma et al. (2017)’s study, in which sex-related differences in VOT did not emerge until adolescence. Interestingly, several studies also suggest that English-speaking adults vary VOT patterns according to speaker sex in a similar way as Mandarin-speaking adults do (Morris et al., 2008; Robb et al., 2005; Ryalls et al., 1997; Swartz, 1992), such that English-speaking women produce stop consonants with longer VOT than men. However, Morris et al. (2008) did not find such a difference in VOT in English-speaking women vs. men, and Robb et al. (2005) only found the sex difference in voiceless aspirated stops, so the robustness of this sex-differentiating pattern in English speakers is still inconclusive. Meanwhile, developmental research in English indicates that sex differences in VOT are not present in five-year-old speech but can be found during adolescence (Koenig, 2000; Whiteside et al., 2004; Whiteside & Marshall, 2001). The emergence of sex differences in VOT production reported in studies of English and Mandarin suggests that this difference could be, at least in part, grounded in the sex-dimorphism differences in oropharyngeal anatomy that do not emerge until puberty. Various anatomical accounts have been offered in previous research. For instance, it has been proposed that the larger supraglottal cavity in men increases the transglottal pressure and thus allows for an earlier vocal cord adduction than in women (Ma et al., 2017; Swartz, 1992; Whiteside & Marshall, 2001). The reduced transglottal pressure in female stop production is also associated with a female characteristic posterior glottal opening (“chink”) in the vocal cords, which leaks air into the supralaryngeal cavity during voicing (Bless & Abbs, 1983; Titze, 1989). Furthermore, Koenig (2000) and Whiteside et al. (2004) pointed out that the stiffer vocal folds in females may be responsible for the later onset of voicing compared to males. It is worth noting that all these proposed mechanisms are speculations that await rigorous physiological studies that link sex-differentiating VOT patterns with anatomical structural development directly. Furthermore, more studies are needed to ascertain the sex-related differential pattern in VOT throughout child development in both languages.

In addition to sex-related anatomical differences, contributions from sociophonetic factors cannot be ruled out. There exists ample research demonstrating stylistic differences between men and women (Stuart-Smith, 2007; also see Simpson, 2009, for a review). Women were found to produce speech more clearly and with better intelligibility (Bradlow et al., 1996; Byrd, 1994; Kwon, 2010). Therefore, the longer VOT in women’s speech could also reflect a careful speech style that enhances speech intelligibility.

Regardless of the origin of the sex-differentiated VOT patterns in Mandarin and in English, the current study represents a case of a relative delay in acquiring socio-indexical knowledge in comparison to the acquisition of lexical-based phonological categories. This finding is at odds with what Li (2017) reported on Mandarin children’s acquisition of voiceless sibilant fricatives. In that study, girls were found to produce a more anterior sibilant fricative /ɕ/ than boys before age 5. The fronting of /ɕ/ in women is a socially marked linguistic phenomenon in Mandarin Chinese and has been termed the “feminine accent” (Cao, 1986; Hu, 1991). Important to note is that Li (2017) found that children produce this sex-related difference in /ɕ/ as early as age 3, and the difference emerges at the same time when children learn to lexically distinguish /ɕ/ from the other two voiceless sibilants, /s/ and /ʂ/. One possible reason for the diverging results between the current study and Li (2017) is that the sex-related difference in voicing contrast in Mandarin Chinese is less pronounced than the fronting of /ɕ/. Instead of affecting a single phoneme, sex-related VOT difference involves both elongating the VOT for voiceless unaspirated stops and shortening the VOT for the voiceless unaspirated stops to create a greater contrast between the two types of sounds. Since this type of sex difference in speech productions affects the relational distance between two contrastive categories, it is potentially more complex for children to master than the fronting of a single consonant as in the case of /ɕ/. Another possibility could be that the sex-differential productions of /ɕ/ are more “social” in nature and less bound by anatomical maturation than stop production; therefore, they can be acquired earlier in life. The conflicting result of the current study and that of Li (2017) suggests that the pace of acquiring socio-indexical markers is not uniform across different sound types. These markers may hinge upon the nature of the indexicality, as well as the degree to which biological and social factors contribute to the patterning of these markers.

Last but not least, the current study demonstrates a clear “overshoot” phase in the mean VOT values together with the measure of *between-categories distance*. Children, as early as two-year-olds, produce the voiceless unaspirated and voiceless aspirated stops with greater VOT values. Because the extent of “overshoot” is more extensive for voiceless aspirated stops than for voiceless unaspirated stops (see Table 1), children also exhibit greater separation between the contrastive stops than adults. In addition, neither the mean VOT values (especially for the voiceless stops) nor the *between-categories distance* reach adult norms by age 5. This finding indicates that the stage of “overshoot” is likely to continue into later years of childhood.

Despite the observation of “overshoot” in the measure of *between-categories distance*, “overshoot” was not found in the other three measures of robustness for phonological contrast (see Table 2). For the measures *%CP*, *within-category dispersion* and *between-categories distance*, consistent and linear development were found over successive age groups towards adulthood. It is important to note that all three measures that did not show “overshoot” take into consideration intra-speaker variability, which is inversely correlated with skillful coordination of vocal gestures (Ferguson & Farwell, 1975; Goodell & Studdert-Kennedy, 1993; Kent, 1976). Therefore, these results collectively suggest that the apparent “overshoot” does not indicate a clearer or more robust distinction in making voicing contrast but may instead reflect an effort to accommodate the greater intra-speaker variability and the associated temporal coordination demand that children have yet to learn to control. Taken together, the four measures of “robustness of phonological contrast” are able to phonetically quantify VOT distributions and development in Mandarin children’s stop productions. The characterization of these four measures reveals both an “overshoot” phase on the surface and the immature gestural coordination under the surface that accompanies the phenomenon of “overshoot”.

With “overshoot” being found in both English- and Mandarin-speaking children’s VOT production, it is tempting to propose that “overshoot” could be a common developmental stage to be found in children who acquire stop consonant systems involving voiceless aspirated stops, regardless of the specific language they speak. One piece of evidence is from a study investigating children’s voiceless stop productions in European Portuguese (Brinca et al., 2016). Although “overshoot” is not the focus of that study and no adult data were reported, the results examining three groups of children aged 5 to 10 demonstrated a clear age-related decrease in VOT values. Future studies on other languages are needed to determine whether “overshoot” is a universal stage that children all go through when acquiring contrasts involving voiceless unaspirated and voiceless aspirated stops.

It is also of interest to explore the basis of “overshoot” in children’s VOT production. Green et al. (2000) pointed out that excessive displacement in early articulatory movement may be an indication of a more general characteristic of immature motor control. They further related “overshoot” in speech activity to that of manual movements, for which overshooting is a feature of immature grasping (Jeannerod, 1988).

Finally, the current study is limited in several ways. First, the observed developmental trajectory and the conclusion of the “overshoot” phase are based on a cross-sectional study. Whether the same pattern applies to each individual child is a question that awaits a true longitudinal study. Second, the present study only focuses on a small window of development, namely ages from 2 to 5 years. It therefore is unable to address the question of whether Mandarin is similar to other languages in that the voiceless unaspirated stops are the “default” or “anchor” category from which the voiceless aspirated category emerges. Since the youngest age examined by the current study is 2 years old, by which time a clear phonological contrast has already been established, younger children need to be studied to confirm this prediction. Nevertheless, the current study sets the stage for future studies by adding to the growing literature on VOT development in diverse languages and also sheds light on children’s lingual motor control development as well as the organization of phonological and phonetic structures during the process of speech sound acquisition.

## 5. Conclusions

In conclusion, this study has demonstrated that Mandarin-speaking children are able to acquire the phonological distinction between voiceless unaspirated and voiceless aspirated lingual stops by 2 years of age, but the phonetic implementation aspect, as evidenced by the acoustic measure voice onset time, takes much longer to develop. Similar to English-speaking children reported in earlier research, Mandarin-speaking children experience a phase of “overshoot” in this phonetic learning process, which suggests a common motoric basis shared by children across these two languages when acquiring lingual stop consonants. Additional measures further suggest that this apparent “overshoot” phase is, in essence, due to greater intraspeaker variability, which in turn reflects children’s immature articulatory motor control.

## Figures and Tables

**Table 1:** Age and gender breakdown of participants. The mean and standard deviation of age (in months) are specified for each group.

	2-Year-Olds (M = 30 Months; SD = 3 Months)	3-Year-Olds (M = 42 Months; SD = 3 Months)	4-Year-Olds (M = 54 Months; SD = 4 Months)	5-Year-Olds (M = 66 Months; SD = 3 months)
Females	14	13	12	12
Males	10	15	11	13
Total	24	28	23	25

**Table 2:** Definitions and interpretations of the four *robustness of contrast* measures.

*Measure*	*Definition*	*Interpretation*
%CP	The percentage of a child’s VOT production that can be correctly predicted by a mixed-effects logistic regression model. It is built on all children’s production available in a dataset. This measure can be calculated for each pair of stops that contrast in voicing (i.e., one measure for /t/-/d/ and one for /k/-/g/).	It indicates how much overlap exists between two contrastive voicing categories.
Within-category dispersion	The mean of the standard deviation of the VOT distribution of voiceless unaspirated and voiceless aspirated stops.	It indicates, on average, how compact or diffuse the shape of VOT distribution of two contrastive voicing categories are.
Between-categories distance	The difference between the means of the two VOT distributions for voiceless unaspirated and voiceless aspirated stops.	It indicates how close or distant the two categories are distributed along the VOT continuum.
Discriminability score	The between-categories distance divided by the square root of the mean of the variances of the two VOT distributions.	It indicates how discriminable the two VOT distributions are.

**Table 3:** Mean (standard deviation) of VOT values (in milliseconds) for each target consonant and for each of the four age groups of children and adults, separated by speakers’ sex.

		/t/	/d/	/k/	/g/
2-year-olds	Female	127.2 (38.9)	61.7 (35.3)	112.6 (35.8)	50.6 (32.1)
	Male	109.3 (24.8)	43.8 (22.0)	108.2 (25.0)	39.2 (23.9)
	All	119.8 (34.3)	54.3 (31.3)	110.8 (31.2)	45.9 (28.9)
3-year-olds	Female	119.7 (16.2)	24.4 (10.1)	116.1 (22.9)	28.7 (10.1)
	Male	128.8 (27.2)	30.0 (14.0)	122.4 (19.8)	34.2 (16.1)
	All	124.6 (22.8)	27.4 (12.4)	119.5 (21.1)	31.6 (13.8)
4-year-olds	Female	121.0 (27.8)	23.4 (22.3)	123.4 (23.1)	23.6 (11.4)
	Male	105.4 (19.4)	19.6 (9.9)	115.6 (21.8)	21.8 (10.7)
	All	113.5 (26.7)	21.6 (17.3)	119.7 (22.3)	22.7 (10.8)
5-year-olds	Female	100.3 (12.6)	19.0 (9.3)	107.6 (19.4)	21.6 (8.9)
	Male	117.4 (11.3)	22.5 (10.4)	106.0 (17.1)	32.6 (21.8)
	All	109.2 (22.0)	20.8 (9.8)	106.8 (17.9)	27.3 (17.5)
Adults	Female	94.0 (12.6)	12.4 (2.4)	90.6 (11.3)	22.5 (3.2)
	Male	77.9 (11.3)	19.1 (6.8)	79.2 (9.7)	31.5 (10.7)
	All	85.7 (14.1)	15.8 (6.1)	84.9 (11.8)	27.0 (9.0)

**Table 4:** Mean (and standard deviation) of the four measures of *robustness of phonological contrast* using VOT for /t/-/d/ contrast and /k/-/g/ contrast produced by children of the four age groups and by adults.

		Robustness of Phonological Contrast Measures			
		%CP (Percentage of Tokens Correctly Predicted by Mixed Effects Models)	Within-Category Dispersion (ms)	Between-Categories Distance (ms)	Discriminability Score
/t/-/d/ contrast	2-year-olds	0.79 (0.41)	44.68 (21.64)	65.47 (33.86)	1.60 (0.98)
	3-year-olds	0.94 (0.24)	32.19 (13.58)	97.14 (25.44)	3.32 (1.71)
	4-year-olds	0.92 (0.23)	26.29 (14.35)	91.93 (22.66)	3.78 (1.93)
	5-year-olds	0.97 (0.18)	21.14 (10.44)	88.36 (17.72)	4.57 (2.44)
	Adults	0.99 (0.11)	10.10 (4.60)	69.93 (16.75)	8.24 (6.66)
/k/-/g/ contrast	2-year-olds	0.80 (0.40)	37.21 (22.36)	64.90 (34.70)	2.10 (1.43)
	3-year-olds	0.92 (0.28)	27.82 (11.53)	87.85 (23.08)	3.45 (1.55)
	4-year-olds	0.96 (0.19)	22.54 (10.35)	96.92 (18.68)	4.56 (2.25)
	5-year-olds	0.96 (0.21)	21.57 (8.73)	79.42 (16.83)	3.95 (1.79)
	Adults	0.98 (0.15)	10.98 (4.35)	57.89 (15.17)	5.77 (2.92)

## Data Availability

Data cannot be shared publicly because of the ethics regulation. Data are available from the corresponding author for researchers who meet the criteria for accessing de-identified data.

## Appendix B. The Word List for the Lingual Stops

		***Target Consonant***			
		**/t/**	**/d/**	**/k/**	**/g/**
Vowel context	/i/	/t^h^i.tsi/	/tiŋ.tsi/		
		梯子 “ladder”	钉子 “nail”		
		/t^h^iŋ.tsi/	/ti.t^h^u/		
		亭子 “pavilion”	地图 “map”		
	/a/	/t^h^ao.tsi/	/ta. ɕaŋ/	/k^h^an.piŋ/	/kao.lou/
		桃子 “peach”	大象 “elephant”	看病 “seeing a doctor”	高楼 “high-rise building”
		/t^h^aŋ. k^h^ai /	/tan. kao/	/k^h^a. t^h^ʂə/	/kaŋ.pi/
		糖块 “candy”	蛋糕 “cake”	卡车 “truck”	钢笔 “pen”
	/u/	/t^h^u.tsi/	/tu. tsi /	/k^h^un.lə/	/ku.tou/
		兔子 “rabbit”	肚子 “belly”	困了”sleepy”	骨头 “bone”
		/t^h^u.tou/	/tun. tʂə/	/k^h^u. tsi/	/kun. tsi/
		土豆 “potato”	蹲着 “squatting”	裤子 “pants”	棍子 “wooden stick”
